# Digital Health Interventions Supporting Recovery for Intensive Care Patients and Their Family Members: A Scoping Review

**DOI:** 10.1016/j.mcpdig.2024.11.006

**Published:** 2024-11-30

**Authors:** Elke Berger, Carola Schol, Sabrina Meertens-Gunput, Dorien Kiers, Diederik Gommers, Louise Rose, Margo van Mol

**Affiliations:** a Department of Intensive Care Adults, Erasmus MC, University Medical Center Rotterdam, The Netherlands; b Department of Intensive Care, Franciscus Gasthuis & Vlietland, Rotterdam, The Netherlands; c Department of Business Intelligence, St. Antonius Hospital, Utrecht, The Netherlands; d Division of Digital Health and Applied Technology Assessment, Florence Nightingale Faculty of Nursing, Midwifery and Palliative Care, King’s College London, London, United Kingdom

## Abstract

Digital innovation in interventions to promote recovery for intensive care unit (ICU) patients and their family members holds promise for enhancing accessibility and improving physical, psychological, and cognitive outcomes. This scoping review provides a comprehensive overview of digital health interventions designed to support the recovery of ICU patients and their family members described in peer-reviewed publications. We searched 6 databases (inception to September 2023); 2 reviewers independently screened citations against predefined eligibility criteria and extracted data. We screened 3485 records and identified 18 original studies and 8 study protocols with a range of study designs published between 2016 and 2023. Most (n=15) completed studies recruited patients only. Digital interventions were delivered through applications, virtual reality, videoconferencing, and smartwatches. In the completed studies, outcomes are described as feasibility, intervention efficacy, or both. Digital interventions supplemented with professional support and personalized feedback were more feasible than self-directed interventions. Further research is essential to ascertain the efficacy and cost-effectiveness of digital interventions in improving outcomes for ICU survivors and their family members.

**Article Highlights**

• This article provides an overview of 18 completed studies and 8 study protocols of digital interventions designed to support the recovery of intensive care unit patients and their family members both during and after intensive care unit admission.
• Most studies focused on only 1 domain of postintensive care syndrome recovery—psychological, physical, or cognitive— and were feasibility studies with mainly preliminary exploration of efficacy outcomes.
• Barriers and facilitators, systematically mapped, were found in 3 themes: patient-centered considerations, technological accessibility and usability, and organization and funding.
• Future studies should evaluate the efficacy of interventions through adequately powered randomized controlled trials while also examining cost-effectiveness and how to optimally personalize them to individual patient needs.

## Background

Critical illness has an important and long-term impact on health-related outcomes of intensive care patients and their family members including physical, psychological, and cognitive well-being.^1^, ^2^, ^3^ These outcomes have been described extensively and are collectively known as postintensive care syndrome (PICS) among survivors and postintensive care syndrome–family (PICS-F) in family members.^4^ Family includes any family member, friends, close relationships, and informal caregivers. Without high quality care after an intensive care unit (ICU) admission, the symptoms of PICS and PICS-F may go unrecognized and untreated.^4^ In many health care systems, multiprofessional integrated teams provide ICU follow-up services.^5^, ^6^, ^7^ These services include in-person interventions, such as informational and peer support, in-hospital follow-up after ICU discharge, and ICU outpatient clinics.^8^^,^^9^ The ICU follow-up might offer valuable care for vulnerable ICU survivors, who are individuals at an increased risk for physical, psychological, or social challenges with a negative impact on their quality of life. However, in-person follow-up interventions may be challenging to access during recovery due to residual disability after critical illness and limitations of pre-existing illnesses.^7^ Because of the flexibility offered by digital technologies, individuals may be able to self or co-manage their recovery at a time and place that suits them best.^10^ Hence, digital interventions commenced both during the ICU admission and in post-ICU follow-up services have the potential to enhance recovery outcomes.

In general, digital health technologies offer benefits in terms of reducing inefficiencies, limiting costs, and offering a more personalized approach to health care access.^11^ Digital technologies, encompassing both hardware (such as smart phones and virtual reality devices) and software (including social media, mobile applications, and web-based programs) have the potential to provide better access to reliable and well-structured health information. In addition, they can support self-monitored recovery and well-being.^11^ Virtual reality (VR) is also a promising tool to help recovery in both physical and psychological domains.^12^, ^13^, ^14^ During the coronavirus disease 2019 pandemic, new digital technologies emerged because of the necessity of alternative ways of offering inter-human contact and support such as virtual visiting^15^ and virtual ICU follow-up service clinics.^16^ However, ICU survivors may be limited in their use of digital interventions due to physical or cognitive inabilities in the recovery phase of a critical illness.^17^^,^^18^ Not all digital health technologies may be suitable for the capabilities of these particular individuals. Following promising digital developments and expected benefits, ICU teams now have an opportunity to consider digital innovation as a method to offer recovery services to ICU patients and their family members.^19^^,^^20^

For any digital intervention to be effective, successful implementation depends on a strategy that takes barriers and facilitators into account. Identifying such factors is crucial, as barriers experienced by the patient, due to insufficient technological access or unsupportive organizations, can influence the effect of any digital solution. Conversely, facilitators can considerably enhance the intervention’s impact. By systematically exploring these elements, we can better inform the design and implementation strategies of digital health interventions, ultimately improving patient and family outcomes during intensive care recovery.^21^^,^^22^

Currently, there is a lack of a comprehensive overview of digital health interventions aimed at supporting the recovery of ICU patients and their family members, both during and after ICU admission. Others have described the development of digital solutions in ICU follow-up; however, these lack a systematic approach,^23^ only address telemedicine for monitoring ICU patients,^24^ or use of a digital intervention only during ICU admission.^13^^,^^19^^,^^25^ This current scoping review will provide an overview in a methodologically systematic manner describing digital interventions used during and after an ICU admission.

The aim of this scoping review was to compile an extensive overview of digital health interventions designed to support recovery of ICU patients and their family members both during and after ICU admission. Our specific objectives were to (1) map elements of the digital health interventions and context of their delivery; (2) describe outcomes reported and how these were measured; (3) provide a narrative summary of the main findings; and (4) explore barriers and facilitators to the delivery of the identified digital interventions.

## Methods

Before commencing this review, one reviewer (M.v.M.) performed an initial search of the international Prospective Register of Systematic Reviews and Google Scholar to identify reviews on this topic thus, avoiding unintentional duplication. We selected to conduct a scoping review as this method allows efficient mapping of available evidence in the domain of target.^26^ We followed the 6-stages framework proposed by Arksey and O’Malley,^27^ advanced by Colquhoun et al^28^ and further expanded by Tricco et al.^29^ Our protocol was registered prospectively with the Open Science Framework (July 23, 2022) with subsequent modification on March 27, 2023 (https://osf.io/z2pk6/). We performed and reported this scoping review in accordance with the Preferred Reporting Items for Systematic Reviews and Meta-Analyses extension for Scoping Reviews.^30^

### Search Strategies

An exhaustive search strategy was developed by an experienced information specialist (S.M.) in collaboration with 2 authors (E.B. and M.v.M.). The search method is based on the Prisma-S extension to the Preferred Reporting Items for Systematic Reviews and Meta-Analyses Statement for Reporting Literature Searches in Systematic Reviews.^31^ The search was developed in Embase.com, optimized for sensitivity and then translated to other databases following the method as described by Bramer et al.^32^ Six databases were searched from inception to September 2023 without language filter: Medline ALL, Embase, Web of Science Core Collection, Cochrane Central Register of Controlled Trials, CINAHL, and PsycINFO.

The search strategies for Medline and Embase used relevant thesaurus terms from Medical Subject Headings and Emtree respectively. In all databases, terms were searched in titles, abstracts, and author keywords. The search contained terms for (1) digital health, (2) intensive care, and (3) follow-up. Terms were combined with Boolean operators AND and OR and proximity operators were used to combine terms into phrases. The full search strategies of all databases are available in Supplemental File 1 (available online at https://www.mcpdigitalhealth.org/). The searches in Embase and Web of Science were limited to exclude all conference papers and protocols from 2019 and older. In all databases, studies only including children were excluded. No study registries were searched, but Cochrane Central retrieves the contents of ClinicalTrials.gov and World Health Organization’s International Clinical Trials Registry Platform. The reference lists of relevant review articles and the included articles in this review were visually scanned for references missed by the search. No authors or subject experts were contacted, and we did not browse unindexed journals in the field. After the search, all identified records were transferred to EndNote X9^TM^,^33^ and duplicates were removed (Supplemental File 1).

### Study Selection Procedure

Two reviewers (E.B. and C.S.) independently screened titles and abstracts based on the inclusion and exclusion criteria. The inclusion criteria adhered to a patient-intervention-control-outcome framework. The population included adult ICU patients, adult ICU survivors, and adult family members or close friends of patients admitted to or surviving an ICU admission. Eligible interventions were digital or e-health solutions aimed at promoting recovery from critical illness, initiated either during ICU admission or after ICU discharge, or interventions providing psychological and informational support for family members during or after ICU admission. Interventions used during ICU admission were included only if they were continued after hospital discharge. Comparators could include usual care, other digital interventions, or no comparator at all, such as in case series or qualitative studies. Outcomes were unrestricted and included any reported results. All quantitative and qualitative study designs were eligible, and peer-reviewed studies published in any language and at any time were included. Studies were excluded if they focused on digit al interventions limited to passive education or information, such as websites or e-learning modules, or if they were categorized as gray literature. Full text versions of eligible studies were retrieved and reviewed by 2 independent reviewers in pairs (E.B., C.S., and M.v.M.) to confirm final inclusion.^34^ We adopted an iterative process and made choices according to new insights during the review process.^35^ The iterative aspect during the review process included extensive team discussions and well-considered decisions. For example, after full text reading, we refined eligibility with new insights on reported interventions not going beyond dissemination of information on a website. In this cycle of going back and forth, we kept in mind the robustness of study methods for a scoping review. Discrepancies were discussed with a fourth reviewer (L.R.) and resolved by consensus.

### Data Charting, Summarizing and Reporting

After agreement on inclusion, 3 reviewers (E.B., C.S., and M.v.M.) independently extracted and collated the following data: author(s), publication year, study design, target population, intervention type, time points for delivery and digital intervention duration, by whom the intervention was delivered, targeted health domains, patient and public involvement, outcomes and their measures, and summarized results. Study protocols were collected for completeness, but results cannot be reported. Results are reported quantitatively with a narrative summary. Barriers and facilitators were organized to the domains of the consolidated framework for implementation research (CFIR).^36^ During regular meetings, 4 reviewers (E.B., C.S., M.v.M., and L.R.) iteratively considered how included study findings related to our scoping review aim and objectives to decide how best to present and summarize with discussion also focusing on implications for future research and clinical practice.

## Results

**Figure 1:**
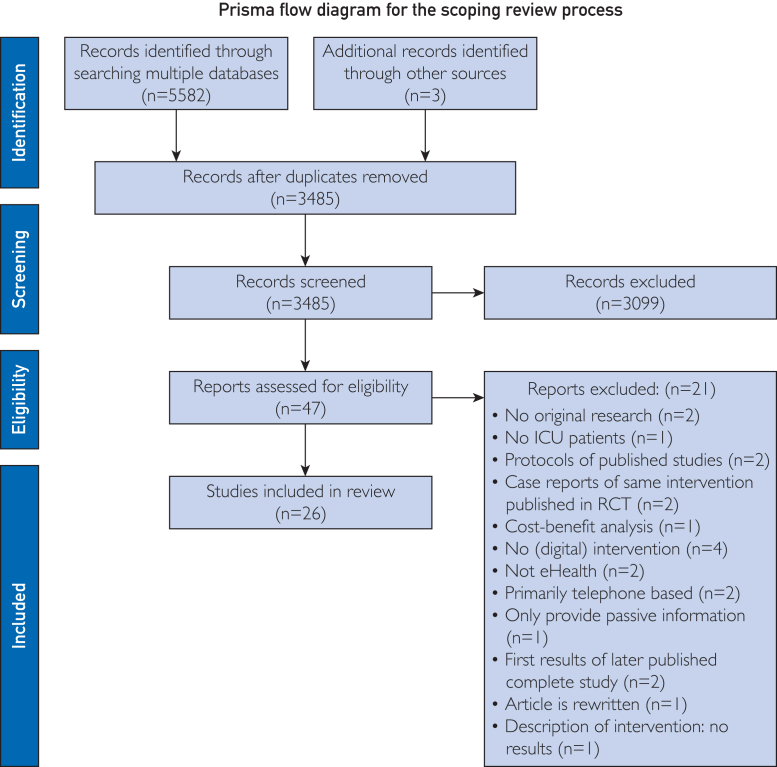
Study selection. ICU, intensive care unit; PRISMA, Preferred Reporting Items for Systematic Reviews and Meta-Analyses; RCT, randomized controlled trial.

The database search identified 5582 records; 3 additional papers were found through reference list screening. After duplicate removal, we screened 3485 records against the inclusion and exclusion criteria, resulting in 47 papers assessed as full text for eligibility. Of these, 26 publications were included.^37^, ^38^, ^39^, ^40^, ^41^, ^42^, ^43^, ^44^, ^45^, ^46^, ^47^, ^48^, ^49^, ^50^, ^51^, ^52^, ^53^, ^54^, ^55^, ^56^, ^57^, ^58^, ^59^, ^60^, ^61^, ^62^ The selection procedure is presented in Figure 1.

### Study Characteristics

We included 18 completed studies and 8 study protocols. Characteristics are presented in Table 1.^37^, ^38^, ^39^, ^40^, ^41^, ^42^, ^43^, ^44^, ^45^, ^46^, ^47^, ^48^, ^49^, ^50^, ^51^, ^52^, ^53^, ^54^, ^55^, ^56^, ^57^, ^58^, ^59^, ^60^, ^61^, ^62^ All studies were published between 2016 and 2023, with 22 (85%) published after 2018. Studies were conducted in Europe (n=12, 46%),^37^^,^^44^, ^45^, ^46^, ^47^^,^^51^^,^^52^^,^^54^^,^^56^, ^57^, ^58^, ^59^ North America (n=10, 38%),^38^, ^39^, ^40^, ^41^^,^^48^^,^^49^^,^^53^^,^^55^^,^^61^^,^^62^ Asia (n=3, 12%),^42^^,^^50^^,^^60^ and Australia (n=1, 4%).^43^ The 18 completed studies,^38^, ^39^, ^40^^,^^42^^,^^44^, ^45^, ^46^^,^^48^^,^^50^, ^51^, ^52^, ^53^, ^54^, ^55^, ^56^, ^57^^,^^60^^,^^62^ had various study designs, including 5 randomized controlled trials (RCTs),^42^^,^^51^^,^^54^^,^^55^^,^^60^ 3 pilot or feasibility RCTs,^38^, ^39^, ^40^ and 4 nonrandomized feasibility or pilot studies.^45^^,^^48^^,^^54^^,^^62^ All 8 study protocols^37^^,^^41^^,^^43^^,^^47^^,^^49^^,^^58^^,^^59^^,^^61^ were RCTs, 2 of which were follow-up studies after pilot studies included in this review.^41^^,^^49^ Patient and public involvement in the development of the intervention was documented in 9 studies and protocols.^40^^,^^43^^,^^47^^,^^51^, ^52^, ^53^, ^54^^,^^57^^,^^59^ In addition, 3 studies incorporated feedback from their own previous research.^39^^,^^41^^,^^49^ Patient and public involvement in the design of the study was documented in 3 studies.^46^^,^^47^^,^^51^

**Table 1:** Characteristics of the Completed Studies and Study Protocols

Reference (y)/setting	Study design	e-health intervention	Intervention led by	Targeted (health) domains	Patient and public involvement
Completed studies					
Patients					
Capin et al,^38^ 2022, USA	Single center pilot randomized feasibility study	Individual bio behaviorally informed, application facilitated, multicomponent telerehabilitation sessions through videoconferencing	Physiotherapist and self-directed	Physical	Patient and public were not involved
Cox et al,^39^ 2019, USA	Multicenter pilot RCT	Application and web-based self-directed mindfulness program (LIFT1)	Psychologist and self-directed	Psychological	Not described, based on Cox 2018; patients were involved in study design and intervention development
Cox et al,^40^ 2023, USA	Single center pilot RCT	Self-guided coping skills training by a native mobile application platform (Blueprint)	Psychologist and self-directed	Psychological	Patients were involved in intervention development
Dong et al,^42^ 2022, China	Single center RCT	Cognitive rehabilitation training through digital operating system (multiscreen touch technology, VR, and man-machine interaction technology)	Self-directed, rehabilitation therapist, psychiatrist, and psychometrist	Cognitive	Not described
Gerber et al,^44^ 2019, Switzerland	Single center nonrandomized trial	VR cognitive stimulation	Study nurse	Cognitive and psychological	Not described
Howroyd et al,^45^ 2023, United Kingdom	Single center service evaluation	Virtual exercise class by videoconferencing followed by a support group	Nurse and a physiotherapist	Physical and psychological	Not described
Hunter et al,^46^ 2022, United Kingdom	Multicenter observational study	Smartwatch and application with or without addition of a multidisciplinary team	Self-directed, physician, physiotherapist, and occupational therapist	Physical	Patients were involved in study design
Ramalingam et al,^50^ 2020, Singapore	Case report	Rehabilitation through applications and videoconferencing	Self-directed, rehabilitation physician, physiatrist, physical therapist, and medical social worker	Physical and psychological	Not described
Rose et al,^52^ 2022, United Kingdom	Early stage innovation report	ICU digital recovery pathway focused on recovery goal setting through e-health platform	ICU recovery coordinator	Physical, cognitive and psychological	Patients and family members were involved in design intervention
Strömberg et al,^54^ 2020, Sweden	Pilot study	Kundalina tele-yoga through videoconferencing	Yoga instructor	Psychological and physical	Patient organization was involved in development, initial testing and modifications of the intervention
Taylor et al,^55^ 2022, USA	Multicenter pragmatic RCT	Virtual delivered multicomponent sepsis transition and recovery program	Nurse navigator	Physical	Not described
Tsavourelou et al,^56^ 2016, Cyprus	Single center eligibility study and cost-benefit analysis	Service oriented pilot platform developed to support tele supervised rehabilitation programs through videoconferencing	Physician, nurse, ergo-physiologist, physiotherapist and psychologist	Physical	Not described
Vlake et al,^57^ 2022, Netherlands	Multicenter RCT	COVID specific ICU-virtual reality	Follow-up clinic nurse or physician	Psychological	Patients and public were not involved in the design, conduct, reporting, or dissemination plans. A former ICU patient was involved in the development of the intervention
Wang et al,^60^ 2022, China	Multicenter randomized study	Virtual reality-based intensive psychological intervention	Self-directed	Psychological	Not described
Wilson et al,^62^ 2018, USA	Single center pilot feasibility study	Computerized cognitive rehabilitation “brain exercises”	Self-directed	Cognitive	Not described
Patients and relatives					
Scruth et al,^53^ 2017, USA	Qualitative description	Electronic ICU diary	Nurse	Psychological	Patients and family members feedback was asked on the application
Reck et al,^51^ 2023, Germany	Re-analysis RCT location independent	Internet-based, therapist-led partner assisted cognitive-behavioral writing therapy	Psychologist	Psychological	Representatives organization German sepsis Aid were asked to comment on the concept of the study and intervention
Family members					
Petrinec et al,^48^ 2021, USA	Single center cohort feasibility study	Application for cognitive-behavioral therapy	Self-directed	Psychological	Not described
Study protocols					
Patients					
Bates et al,^37^ 2020, United Kingdom	Single center feasibility RCT	Videoconference appointments and online eye movement desensitization and reprocessing sessions	Psychotherapist	Psychological	Not described
Cox et al,^41^ 2020, USA	Multicenter RCT	Mobile mindfulness application and web-based; improving application’s (LIFT2)	Therapist or digital	Psychological	Not described, based on Cox 2019
Ewens et al,^43^ 2020, Australia	Single center RCT	Web-based intensive care recovery program ICU together	Researchers	Psychological	The intervention was tested by ICU survivors
McGregor et al,^47^ 2021, United Kingdom	Multicenter RCT	Online home-based, supervised, group rehabilitation program and psychological support	Clinical exercise physiologist/physiotherapist	Psychological and physical	The patient and public group will advise on intervention content, study processes, and outcomes
Vlake et al,^59^ 2022, Netherlands	International multicenter RCT	ICU-specific VR (HORIZON-IC)	Study team	Psychological	Former ICU patients were involved in the development of the intervention. Patients or the public were not involved in the design, or conduct, or reporting, or dissemination plans
Wang et al,^61^ 2018, USA	Multicenter RCT	Computer-accessed online cognitive training modules and physical exercises through video conference	Self-directed and a trained facilitator	Cognitive	Not described
Family members					
Petrinec et al,^49^ 2021, USA	Single center randomized pilot study	Smartphone application with a suite of tools based on cognitive behavioural therapy and mindfulness principles	Self-directed	Psychological	Based on feedback of study by Petrinec et al,^49^ 2021
Vlake et al,^58^ 2021, Netherlands	Multicenter RCT	ICU-virtual reality-family in hospital or link to digital VR at home	Study team	Psychological	Patients or the public were not involved in the design, conduct, reporting, or dissemination plans

ICU, intensive care unit; RCT, randomized controlled trial; VR, virtual reality.

### Elements and Delivery Context of the Digital Interventions

**Figure 2:**
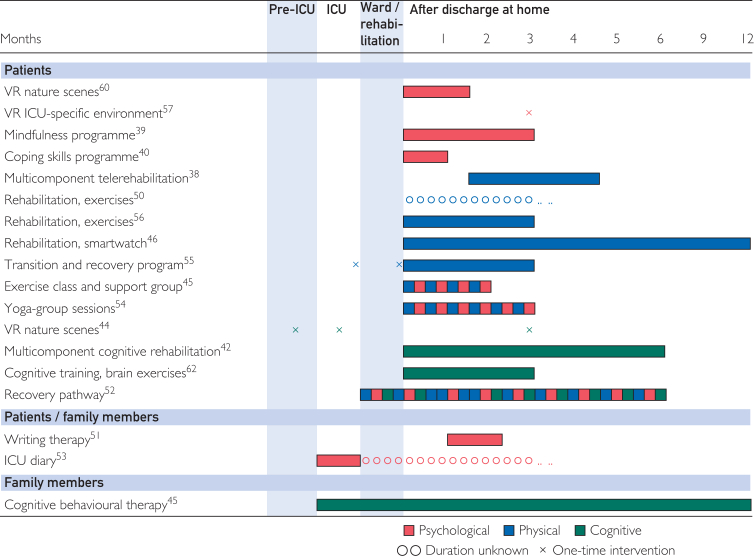
Interventions and targeted health domains for patients and family members. ICU, intensive care unit; VR, virtual reality.

In the 18 completed studies, interventions were delivered by web-based applications (n=14, 78%),^38^, ^39^, ^40^^,^^42^^,^^45^^,^^46^^,^^48^^,^^50^, ^51^, ^52^, ^53^, ^54^^,^^56^^,^^62^ some of which also made use of videoconferencing (n=7, 39%),^38^^,^^45^^,^^50^^,^^52^^,^^54^, ^55^, ^56^ VR (n=4, 22%),^42^^,^^44^^,^^57^^,^^60^ a smartwatch (n=1, 6%),^46^ and an activity tracker (n=1, 6%).^38^ Studies recruited either patients only,^38^, ^39^, ^40^^,^^42^^,^^44^, ^45^, ^46^^,^^50^^,^^52^^,^^54^, ^55^, ^56^, ^57^^,^^60^^,^^62^ both patients and family members,^51^^,^^53^ or only family members.^48^ The interventions were delivered after hospital discharge or delivered both during ICU admission and after hospital discharge (Figure 2). The duration of study participation varied from 14 minutes for a VR intervention^57^ to up to 1 year for an intervention delivered using a smartwatch,^46^ and a cognitive-behavioral therapy application.^48^ Digital interventions were completely self-directed (n=3, 17%),^48^^,^^60^^,^^62^ or partially self-directed (n=6, 33%).^38^, ^39^, ^40^^,^^42^^,^^46^^,^^50^ In the partially self-directed interventions, the patient or family member had some level of autonomy but were also guided by a health care professional. Other interventions were solely provided by a health care professional including a research nurse (n=6, 33%),^44^^,^^45^^,^^53^^,^^55^, ^56^, ^57^ physician (n=4, 22%),^46^^,^^50^^,^^56^^,^^57^ psychologist (n=4, 22%),^39^^,^^40^^,^^51^^,^^56^ physiotherapist (n=6, 33%),^38^^,^^42^^,^^45^^,^^46^^,^^50^^,^^56^ yoga instructor (n=1, 6%),^54^ ICU recovery coordinator (n=1, 6%),^52^ occupational therapist (n=1, 6%),^46^ or a combination of delivery methods (n=8, 44%).^38^, ^39^, ^40^^,^^42^^,^^45^^,^^46^^,^^50^^,^^56^ A complete overview of the elements and delivery context of the digital interventions is presented in Table 1.

### Reported Outcomes and Measures

Nine studies reported process outcomes, including feasibility (n=5, 28%), acceptability (n=5, 28%), adherence (n=5, 28%), usability (n=5, 28%), safety (n=3, 17%), fidelity (n=1, 6%), eligibility (n=1, 6%), and recruitment and retention (n=1, 6%).^38^, ^39^, ^40^^,^^44^, ^45^, ^46^^,^^48^^,^^52^, ^53^, ^54^^,^^56^ Consent rates in the included studies varied between 14.5%^38^ and 75%,^48^ and intervention adherence varied between 70%^46^ and 100%.^54^ Ten studies also reported exploratory efficacy outcomes.^38^, ^39^, ^40^^,^^44^, ^45^, ^46^^,^^48^^,^^52^^,^^54^^,^^60^ Four studies reported efficacy outcomes as a primary outcome.^42^^,^^55^^,^^57^^,^^60^ Efficacy outcomes included those in the cognitive, psychological, and physical domains, or combined outcomes (Figure 2). One study included a cost-benefit analysis calculating that the initial investment into the digital intervention might outweigh the costs of rehospitalization due to unaddressed rehabilitation needs and new complications.^56^

Psychological outcomes included prevalence and severity of psychological distress (posttraumatic stress symptoms, anxiety, and depression), health-related quality of life, mindfulness skills, coping skills, and self-efficacy.^38^, ^39^, ^40^^,^^42^^,^^44^^,^^45^^,^^48^^,^^50^, ^51^, ^52^^,^^54^^,^^57^^,^^60^ Physical outcomes included sleep, exercise capacity, daily step count, walk test function and balance, shoulder disability, grip strength, breathlessness, functional independence, physical symptoms, and nutritional recovery.^38^^,^^39^^,^^45^^,^^46^^,^^50^^,^^52^^,^^54^ Other outcomes included cognitive function,^38^^,^^42^^,^^44^^,^^54^ mortality, and hospital readmission.^55^ A complete overview of reported study outcomes and measurement instruments is provided in Table 2.^38^, ^39^, ^40^^,^^42^^,^^44^^,^^45^^,^^48^^,^^50^, ^51^, ^52^^,^^54^^,^^55^^,^^57^^,^^60^

**Table 2:** Outcomes and Measurement Instruments

Domain	Outcome	Measurement instrument
Process	Feasibility	Feasibility of intervention measure^52^
	Acceptability	Acceptability of intervention measure^52^
		Intervention appropriateness measure^52^
		Adapted client satisfaction questionnaire^39^
	Usability	10-item system usability scale^39^
	Costs	Health care utilization: client services receipt inventory^52^
Psychological	Posttraumatic stress symptoms	Posttraumatic stress scale^39^^,^^40^^,^^60^
		Impact of event scale–revised^52^^,^^57^
		Post traumatic stress disorder (PTSD) checklist for the diagnostic and statistical manual of mental disorders (DSM-5), fifth edition^48^^,^^51^
		Clinician-administered PTSD scale for DSM-5^51^
	Anxiety and depression	Hospital anxiety and depression scale^40^^,^^45^^,^^48^^,^^54^^,^^57^^,^^60^
		Intensive care psychological assessment tool^45^
	Anxiety	Generalized anxiety disorder 7-item scale^39^^,^^52^
	Depression	Patient health questionnaire–2^55^
		Patient health questionnaire–8^38^
		Patient health questionnaire–9^39,50,52,55^
	Emotion/mood	Positive and negative affect schedule^60^
		Cognitive and affective mindfulness scale-revised^39^
	Loneliness	Three-item loneliness scale^38^
	Sleep	Minimal insomnia symptom scale^54^
	Somatic symptoms	Patient health questionnaire–15^39^
	Self-efficacy	Patient reported outcomes measurement and information system (PROMIS) V1.0 general self-efficacy^38^
		PROMIS short form self- efficacy for managing chronic conditions^38^
		Mental health self-efficacy scale^48^
		Brief coping inventory^39^
		Pearlin mastery^52^
		Personal gain scale (caregivers)^52^
	Motivation	Exercise motivation index^54^
	Therapeutic alliance	Therapeutic alliance scale for the assessment of general effectiveness factors in psychotherapy^51^
Physical	Exercise capacity	6-minute walk test,^50^^,^^54^ 30 seconds chair stand test,^38^ time up-and-go test,^38^ and sit-to-stand test^45^^,^^54^
	Function	Clinical frailty scale^38^
		Gait speed test^54^
		Functional assessment of chronic illness therapy- fatigue^52^
		Functional independence measure^50^
		Nottingham extended activities of daily living scale^52^
	Balance	Four-stage balance test^38^
		Activities-specific balance confidence scale^38^
	Shoulder disability	Quick dash upper limb questionnaire^45^
	Breathlessness	Medical research council dyspnea scale^38^^,^^45^
Cognitive	Cognitive	Montreal cognitive assessment^38^^,^^42^^,^^44^^,^^54^
Physical, psychological, social	Health-related quality of life	36-Item short form health survey^42^^,^^57^
		12-Item short form health survey^48^
		Euroqol 5 dimensions^44^^,^^45^^,^^52^^,^^54^^,^^57^
		Quality of life with visual analog scale^39^^,^^40^
	Global health	PROMIS scale V.1.2 global health measure^38^
	Caregiver burden	Zarit burden interview^52^
		Caregiving impact scale^52^
		Caregiving assistance scale^52^

### Narrative Summary of the Main Findings Categorized by Domain

#### Psychological Domain

Most interventions focused on the psychological domain.^39^^,^^40^^,^^51^^,^^53^^,^^57^^,^^60^ Two studies used VR interventions. One used VR to imitate a 3-dimensional ICU, with explanations of different facets of the ICU environment, treatment information, and ICU staff roles. This study did not show a beneficial effect on patient psychological distress.^57^ The other study used VR to show nature scenes and music, reporting an improvement in symptoms of post traumatic stress disorder, anxiety, and depression.^60^ A cognitive-behavioral writing therapy for patients and their partners, consisting of 10 writing tasks during 5 weeks found a decrease in posttraumatic stress symptoms and severity in a per-protocol analysis,^51^ but not in the intention-to-treat analysis.^63^ Unlike studies with primary efficacy outcomes, 2 studies explored the feasibility of self-directed interventions in the psychological domain.^39^^,^^40^ These included a self-directed mindfulness program,^39^ and a self-guided coping skills program.^40^ Both interventions compared a self-directed application with the addition of a therapist^39^ or delivery by phone.^40^ Dropout rates were higher in the group using the self-directed mindfulness mobile application (29%) compared with those randomized to the telephone mindfulness program (10%), and the web-based education program (11%).^39^ The self-guided coping skills program found the highest dropouts in the group without a therapist (18.8% vs 42.9%).^40^ An electronic ICU diary was found to be feasible and usable by family members of ICU patients.^53^ A complete overview of reported results is provided in Supplemental File 2 (available online at https://www.mcpdigitalhealth.org/).

#### Physical Domain

Interventions aimed at the physical domain focused on rehabilitation through exercise,^38^^,^^45^^,^^46^^,^^50^^,^^54^^,^^56^ and one aimed at reducing readmissions and mortality.^55^ Four studies were pilot or feasibility studies;^38^^,^^45^^,^^46^^,^^54^ one RCT,^55^ one description of an intervention,^50^ and one assessment of the number of eligible patients.^56^ All feasibility/pilot studies were considered feasible and safe to use.^38^^,^^45^^,^^54^ Rehabilitation interventions using exercise were delivered individually,^38^^,^^46^^,^^50^ or in a group.^45^^,^^54^^,^^56^ Two interventions included a psychological component, ie a tele-yoga intervention^54^ and an exercise class followed by a support group session.^45^ One physical intervention involved the self-directed use of a smartwatch.^46^ In this multicenter comparison study, use of a smartwatch to monitor step count was compared with smartwatch use with feedback from a remote multidisciplinary team based on the smartwatch data with adaptations made to the rehabilitation plan.^46^ In both groups, daily step counts increased as motivation improved by making recovery progress more visible. The group with feedback from the multidisciplinary team achieved an even higher increase in step counts.^46^ The inability to customize interventions to a patient’s capabilities and requirements may result in nonparticipation, especially among those with poor functional status^38^ or those perceiving themselves as either too ill or too well for the intervention.^45^ An RCT of a virtual, navigator-led platform for a sepsis transition and recovery program, found a lower combined rate of mortality and readmission 30 days after discharge in patients that were randomized to the program.^55^ Of note, only ∼42% of patients were admitted to the ICU. Two other studies included a case description with no results of the digitally delivered part of the intervention at home^50^ and a study that reported the number of ICU patients eligible to participate in the intervention, along with a cost-benefit analysis.^56^ This indicated that the intervention was likely to be cost effective despite the financial burden in the context of the expected health benefits.^56^

#### Cognitive Domain

Interventions targeting the cognitive domain included cognitive rehabilitation training using cognitive exercises,^42^^,^^62^ cognitive stimulation through VR,^44^ and cognitive-behavioral writing therapy for family members.^48^ Two of these studies were feasibility/pilot studies,^48^^,^^62^ one observational study^44^ and 1 RCT.^42^ The RCT investigating cognitive rehabilitation training including cognitive exercises with multiscreen touch technology, VR, and man-machine scene interaction technology, combined with music therapy, aerobic training, and a mental health intervention. This multicomponent program reported improvement in both cognitive function scores and quality of life.^42^ In the study using cognitive stimulation through VR, the intervention was considered acceptable, with patients remembering the VR better than the rest of their ICU stay.^44^ The results of the 2 pilot studies found that the interventions were feasible.^48^^,^^62^ Only one of these interventions was developed specifically to support family members.^48^

#### Combined Domains

Finally, one intervention targeted a combination of psychological, physical, and cognitive domains. This digital care pathway was developed to support postintensive care recovery and achieving goals after critical illness.^52^ Initial evaluation of this pathway indicated that it was feasible to deliver and had good acceptability ratings.^52^

### Barriers and Facilitators to the Delivery of the Digital Intervention

Eleven studies identified 11 barriers and 12 facilitators, as summarized in Table 3^38^, ^39^, ^40^^,^^45^^,^^46^^,^^48^^,^^51^, ^52^, ^53^, ^54^^,^^56^ and organized according to CFIR domains.^38^, ^39^, ^40^^,^^45^^,^^46^^,^^48^^,^^51^, ^52^, ^53^, ^54^^,^^56^ Twelve factors were classified within the innovation domain,^38^, ^39^, ^40^^,^^45^^,^^46^^,^^48^^,^^51^^,^^53^ 4 within characteristics of individuals,^45^^,^^46^^,^^51^ 3 within the inner setting,^40^^,^^45^^,^^51^ 3 within process implementation,^52^^,^^54^^,^^56^ and 2 within the outer setting domain.^52^

**Table 3:** Barriers and Facilitators in CFIR Framework

CFIR-domain	Patient-relative level		Organizational−professional level	
	Barriers	Facilitators	Barriers	Facilitators
Innovation	• Unconventional nature of internet-delivered psychotherapy^51^ • Separate device needed for the intervention^48^	• Ease of use for family members^53^ • Personal feedback from a multidisciplinary team^46^ • Interactive features^39^^,^^46^ • Visualization of progress^39^^,^^46^ • Self-directed therapy^39^ • Availability of a therapist for support^40^ • Easy accessibility^38^^,^^40^	• High costs^38^^,^^53^ • Intervention is not suitable for all kind of patients (to ill, to well)^38^^,^^40^^,^^45^	• Ease of use for ICU staff^53^
Outer setting		• Help from family members^52^ • Tablets available for use^52^		
Inner setting	• A lack of technology access^45^		• Lack of access to potential participants^51^ • Difficult to identify the right patients (level of illness)^40^	
Characteristic of individuals	• Low familiarity with technology, especially in the older generation^52^ • Language barriers^45^^,^^52^ • Lack of motivation^46^ • Poor cognitive ability^52^			
Process of implementation				• Good interprofessional relationships^52^ • Standardizing the intervention^54^ • Manuals and guidelines of use and management of the infrastructure services^56^

Innovation: the characteristics of the intervention or innovation itself, such as its complexity, adaptability, and perceived advantage compared with existing solutions; outer setting: the external environment surrounding the organization, such as patient needs, external policies, incentives, and peer pressure from other organizations; inner setting: the internal environment of the organization, such as its culture, climate, communication processes, and readiness for change; characteristics of individuals: this pertains individuals involved in the implementation, including their knowledge, beliefs, self-efficacy, and attitudes toward the innovation; and process of implementation: the steps and activities involved in implementing the innovation, such as planning, engaging stakeholders, executing the plan, and evaluating the outcomes.

CFIR, consolidated framework for implementation research; ICU, intensive care unit.

## Discussion

We identified 18 completed studies and 8 study protocols, with the majority published in the last 6 years. Although mapping elements of the digital health interventions and context of delivery, we identified various digital tools, including applications, digital platforms, VR, and wearable devices, some used in combination. Delivery of these interventions spans both ICU admission and following ICU, with diverse delivery durations and involvement of health care professionals. Most of the included studies targeted ICU patients few targeted family members.

The number of studies we identified pales in comparison with the extensive body of research exploring digital interventions for other patient groups, such as those with a chronic illness. For example, a recent systematic review included 130 RCTs of digital interventions for managing chronic illness.^64^ The number of studies on family members is even smaller than the number recruiting patients. The limited number of digital interventions targeting family members of ICU patients is concerning given the psychological distress for family members associated with ICU admission.^4^ In comparison, a systematic review of digital health interventions supporting family caregivers of individuals with a diversity of chronic mental and physical illnesses identified 40 studies using various digital health tools.^65^ Remarkably, more than 85% of these studies reported significant improvements in caregiver outcomes.^65^ Therefore, further research is needed evaluating digital interventions to support family members both during and after an ICU admission.

We provide an overview of outcomes evaluated and main findings. Notably, most studies focused on only 1 domain of PICS recovery—psychological, physical, or cognitive. Although this is a reasonable starting point for innovation in follow-up care, future developments would benefit from a more modular recovery pathway that better addresses the diverse range of health-related impairments experienced by PICS survivors. Given the variability in digital interventions and the early stage of their development, no single one-size-fits-all digital solution for supporting patients and their families during and after ICU admission was identified.

Given the relative infancy of research into digital interventions for critically ill patients and family members, most of the studies we identified focused on feasibility with mainly preliminary exploration of efficacy outcomes. Consent rates were variable, ranging from 15% to 75%. However, adherence rates were generally high ie, 70% to 97%. This is in line with a previous systematic review of interventions supporting ICU recovery, which found that interventions offering flexibility in the timing of delivery had better consent and retention rates due to the different recovery trajectories that patients may have.^16^ We anticipate that the flexibility of an intervention, including broad availability in self-management tools, enhances its usability among ICU survivors and family members.

Our exploration of barriers and facilitators identified factors involved in all domains of implementational science. These factors can be grouped into 3 themes: patient-centered considerations; technological accessibility and usability; and organization and funding. Patient-centered considerations take the patient’s individual needs into accounts. We found that interventions tailored to the individual had a higher consent rate and adherence. The importance of personalizing follow-up care for ICU survivors is well-recognized, but it has yet to be widely implemented in ICU aftercare, developed on a larger scale, or incorporated into clinical guidelines.^66^^,^^67^ This shift to a personalized approach needs to address the challenges of a cultural change in the ICU team. Digital follow-up tools, in a combination with a personal consult, inpatient clinics, or peer support, can help to enlarge offerings in a human-centered way of ICU care.^68^ Studies of digital interventions that were completely self-directed had lower adherence than those that incorporated interaction with a health care professional. For instance, in a study of a self-guided coping skills training program through an application, a higher dropout rate was observed in the group without a therapist.^40^ Similarly, a smartwatch study incorporating a multidisciplinary team providing personalized feedback reported a positive impact on the motivation to use the device.^46^ These findings imply that, to ascertain patient-centeredness, the intervention target group must be included in its development from the start.^69^ This was not always the case, as patient and public involvement in the development of the intervention was described in only about half of the included studies.

Technological accessibility and usability are fundamental for the successful implementation and adaptation of digital interventions. The included studies highlight several key factors that influence accessibility. For instance, device choice can considerably impact user engagement. Carrying a second phone was cumbersome for some participants, potentially diminishing their involvement with the application.^48^ In addition, hardware availability is essential. Providing tablets to participants who do not own one enhances accessibility and usability.^52^ Furthermore, sufficient technology support is important for successful implementation. Educating health professionals within a diverse technological infrastructure (regarding issues of services and systems) proved challenging, necessitating the preparation of detailed manuals and guidelines for both infrastructure management and clinical practice.^56^

Finally, some barriers and facilitators were identified in the organizational and funding aspects domain. Twice, intervention costs were identified as a barrier.^38^^,^^53^ Only 1 study reported a cost-benefit analysis. In a systematic review and meta-analysis of digital interventions for the rehabilitation of cardiac care patients, the superiority of digital care was suggested because of its convenience, accessibility, and cost-effectiveness.^70^

### Future Research

Although the studies included in our scoping review have shown the potential of digital innovations in improving the outcomes of ICU survivors and their family members, further research is needed to address key evidence gaps. Future studies should rigorously evaluate the efficacy of interventions through adequately powered RCTs while also examining how to optimally time and personalize them to individual patient needs. The rapid advancements in artificial intelligence and large language models offer exciting opportunities for providing highly tailored, scalable support by leveraging the data-rich ICU environment. Embedding of digital interventions within the health care system and an exploration of cost-effectiveness are rarely described, making this is an important area of further research. Robust health economic evaluations and implementation science research are critical to report value to payers and address multilevel barriers to real-world adoption and sustainability.

### Strengths and Limitations

The broad scope and inclusion of studies, with a wide range of study designs and methodologies included in this review, is a strength. Our transparent review process, following the 6 stages outlined in the Arksey and O’Malley framework, provides rigor to our study.^27^, ^28^, ^29^ Another strength of our review is that we systematically mapped the identified barriers and facilitators to the domains of CFIR. This approach provided a structured and comprehensive way to organize and interpret the findings, ensuring that all relevant factors influencing implementation were captured within a widely recognized framework.

Our review has limitations. Although scoping review experts recommend that gray literature should be explored,^71^ we purposely omitted this, as our focus was on empirical evidence concerning digital interventions, which is rarely found in the gray literature. Therefore, we could have missed digital interventions that are being used but have not been empirically studied or studies that were unable to get their article published in a journal due to publication bias. We decided to exclude any study that reported on digital interventions initiated in the general ward or addressing the pediatric setting. The results of these studies could be relevant for developing innovative digital pathways for ICU survivors and families.

## Conclusion

This scoping review reported on 18 completed studies and 8 study protocols related to diverse digital interventions designed to support the recovery of ICU patients and their family members. Most interventions targeted patients directly; only a limited number were tailored to support family members. Interventions incorporating interaction with health care professionals and personalized feedback on rehabilitation progress appeared to enhance feasibility and adherence rates. Overall, the evolving landscape of the development and application of digital interventions in the ICU recovery pathway underlines their potential in enhancing quality of life for ICU survivors and family members.

## Potential Competing Interests

Dr Rose reports grants or contracts from NIHR and ICS (to the institution); payment or honoraria–Drager Medical speaking fees (no relationship to the topic of this manuscript); and participation on a Data Safety Monitoring Board or Advisory Board for Hamilton Medical DSMB (no relationship to the topic of this manuscript); and acknowledges that she is an author of a study included in the review. Dr van Mol reports support for the present manuscript from Netherlands Organisation for Health Research and Development (ZonMw) by the funding scheme “Personal grant: talent development of nurses with a doctorate”, project number 10040022110001 (employed at Erasmus). The other authors report no competing interests.
