# Supplementary material for: Digital Health Interventions Supporting Recovery for Intensive Care Patients and Their Family Members: A Scoping Review
Source: Mayo Clin Proc Digit Health. 2024 Nov 30;3(1):100185. doi: 10.1016/j.mcpdig.2024.11.006 (PMC11975854; doi:10.1016/j.mcpdig.2024.11.006)
Supplement: Supplementary Data [file mmc1.pdf]

Page 1 – 4: : Supplemental File S1 Search Strategy

Page 5 – 9: Supplemental File S2 Description of the primary results

Supplemental File S1 Search strategy

The search was carried out in the databases Medline ALL via Ovid (1946 to Daily Update), Embase.com (1971-present), Web of Science Core Collection (Science Citation Index Expanded (1975-present); Social Sciences Citation Index (1975-present); Arts & Humanities Citation Index (1975-present); Conference Proceedings Citation Index- Science (1990-present); Conference Proceedings Citation Index- Social Science & Humanities (1990-present) and Emerging Sources Citation Index (2015-present)), PsycInfo via Ovid (1806-present), Cinahl via EBSCO (1982-present) and the Cochrane Central Register of Controlled Trials via Wiley (1992-present). Additionally a search was performed in Google Scholar from which the 200 highest-ranked references were downloaded using the software Publish or Perish[1]. After the original search was performed in May 2022, the search was last updated on 5 september 2023, using the methods as described by Bramer et al[2]. The number of records are described in table 1.

Medline Ovid

(exp Telemedicine/ OR exp Internet/ OR exp Cell Phone/ OR exp Computers, Handheld/ OR exp Computer Simulation/ OR Mobile Applications/ OR (telehealth* OR tele-health* OR telemedicine* OR telemonitor* OR telecare* OR telerehabilitat* OR tele-rehabilitat* OR eHealth OR e-health* OR telerehabilitation* OR ((tele* OR electr*) ADJ3 (medicin* OR monitor* OR care* OR rehabilitation* OR device*)) OR (tele* ADJ3 health*) OR (electr*-health* NOT (electr*-health-record*)) OR mHealth OR m-Health* OR mobile-health* OR internet* OR website* OR digital* OR world-wide-web OR web-based OR webbased OR ((web OR mobile) ADJ3 (application* OR app* OR device*)) OR ((mobile OR cell* OR smart*) ADJ (phone*)) OR smartphone* OR virtual-realit* OR simulat* OR augment-realit* OR (electr* ADJ3 system*) OR ((virtual*) ADJ3 (care* OR communicat* OR technolog*)) OR tablet* OR (electr* ADJ3 decision-aid*) OR smartwatch*).ab,ti,kf.) **AND** (exp Intensive Care/ OR Critical Illness/ OR Critical Care/ OR (ICU OR GICU OR ((intensive OR critical*) ADJ3 (care*)) OR critical*-ill*).ab,ti,kf.) **AND** (exp Aftercare/ OR home monitoring/ OR (aftercare* OR after-care* OR followup* OR follow-up OR ((post OR after) ADJ3 (IC OR ICU OR intensive-care* OR discharge*)) OR postIC OR postICU OR postintensive* OR transition* OR ((ICU OR IC OR intensive-care* OR critical*-ill*) ADJ3 (survivor* OR rehabilitat*)) OR mobili*).ab,ti,kf.) *NOT ((Adolescent/ OR exp Child/ OR exp Infant/) NOT exp Adult/) NOT ((news OR congres* OR abstract* OR book* OR chapter* OR dissertation abstract*).pt. AND 1800:2019.(sa_year))*

Embase.com

('telehealth'/exp OR 'Internet'/exp OR 'mobile phone'/exp OR 'tablet computer'/de OR 'computer simulation'/exp OR 'mobile application'/exp OR (telehealth* OR tele-health* OR telemedicine* OR telemonitor* OR telecare* OR telerehabilitat* OR tele-rehabilitat* OR eHealth OR e-health* OR telerehabilitation* OR ((tele* OR electr*) NEXT/3 (medicin* OR monitor* OR care* OR rehabilitation* OR device*)) OR (tele* NEXT/3 health*) OR (electr*-health* NOT (electr*-health-record*)) OR mHealth OR m-Health* OR mobile-health* OR internet* OR website* OR digital* OR world-wide-web OR web-based OR webbased OR ((web OR mobile) NEAR/3 (application* OR app* OR device*)) OR ((mobile OR cell* OR smart*) NEXT/1 (phone*)) OR smartphone*

[1] *Harzing, A.W. (2007) Publish or Perish, available from https://harzing.com/resources/publish-or-perish*
[2] *Bramer W, Bain P. Updating search strategies for systematic reviews using EndNote. J Med Libr Assoc. 2017 Jul;105(3):285-289.*

OR virtual-realit* OR simulat* OR augment-realit* OR (electr* NEAR/3 system*) OR ((virtual*) NEAR/3 (care* OR communicat* OR technolog*)) OR tablet* OR (electr* NEAR/3 decision-aid*) OR smartwatch*):ab,ti,kw) **AND** ('intensive care'/de OR 'intensive care unit'/exp OR 'critical illness'/de OR 'critically ill patient'/de OR (ICU OR GICU OR ((intensive OR critical*) NEAR/3 (care*)) OR critical*-ill*):ab,ti,kw) **AND** ('aftercare'/exp OR 'home monitoring'/de OR (aftercare* OR after-care* OR followup* OR follow-up OR ((post OR after) NEXT/3 (IC OR ICU OR intensive-care* OR discharge*)) OR postIC OR postICU OR postintensive* OR transition* OR ((ICU OR IC OR intensive-care* OR critical*-ill*) NEAR/3 (survivor* OR rehabilitat*)) OR mobili*):ab,ti,kw) *NOT (juvenile/exp NOT adult/exp) NOT ([conference abstract]/lim AND [1800-2019]/py)*

PsycINFO

(exp Electronic Health Services/ OR exp Internet/ OR exp Mobile Devices/ OR exp Computer Simulation/ OR Mobile Applications/ OR (telehealth* OR tele-health* OR telemedicine* OR telemonitor* OR telecare* OR telerehabilitat* OR tele-rehabilitat* OR eHealth OR e-health* OR telerehabilitation* OR ((tele* OR electr*) ADJ3 (medicin* OR monitor* OR care* OR rehabilitation* OR device*)) OR (tele* ADJ3 health*) OR (electr*-health* NOT (electr*-health-record*)) OR mHealth OR m-Health* OR mobile-health* OR internet* OR website* OR digital* OR world-wide-web OR web-based OR webbased OR ((web OR mobile) ADJ3 (application* OR app* OR device*)) OR ((mobile OR cell* OR smart*) ADJ (phone*)) OR smartphone* OR virtual-realit* OR simulat* OR augment-realit* OR (electr* ADJ3 system*) OR ((virtual*) ADJ3 (care* OR communicat* OR technolog*)) OR tablet* OR (electr* ADJ3 decision-aid*) OR smartwatch*).ab,ti.) **AND** (Intensive Care/ OR (ICU OR GICU OR ((intensive OR critical*) ADJ3 (care*)) OR critical*-ill*).ab,ti.) **AND** (exp Aftercare/ OR (aftercare* OR after-care* OR followup* OR follow-up OR ((post OR after) ADJ3 (IC OR ICU OR intensive-care* OR discharge*)) OR postIC OR postICU OR postintensive* OR transition* OR ((ICU OR IC OR intensive-care* OR critical*-ill*) ADJ3 (survivor* OR rehabilitat*)) OR mobili*).ab,ti.) *AND 300.ag. NOT ((news OR congres* OR abstract* OR book* OR chapter* OR dissertation abstract*).pt. AND 1800:2019.(sa_year))*

CINAHL

(MH Telemedicine OR MH Telerehabilitation OR MH Telehealth OR MH Internet+ OR "World Wide Web"+ OR MH "Cellular Phone"+ OR MH "Computers, Portable"+ OR MH "Computer Simulation"+ OR "Mobile Applications"+ OR TI (telehealth* OR tele-health* OR telemedicine* OR telemonitor* OR telecare* OR telerehabilitat* OR tele-rehabilitat* OR eHealth OR e-health* OR telerehabilitation* OR ((tele* OR electr*) N2 (medicin* OR monitor* OR care* OR rehabilitation* OR device*)) OR (tele* N2 health*) OR (electr*-health* NOT (electr*-health-record*)) OR mHealth OR m-Health* OR mobile-health* OR internet* OR website* OR digital* OR world-wide-web OR web-based OR webbased OR ((web OR mobile) N2 (application* OR app* OR device*)) OR ((mobile OR cell* OR smart*) N1 (phone*)) OR smartphone* OR virtual-realit* OR simulat* OR augment-realit* OR (electr* N2 system*) OR ((virtual*) N2 (care* OR communicat* OR technolog*)) OR tablet* OR (electr* N2 decision-aid*) OR smartwatch*) OR AB (telehealth* OR tele-health* OR telemedicine* OR telemonitor* OR telecare* OR telerehabilitat* OR tele-rehabilitat* OR eHealth OR e-health* OR telerehabilitation* OR ((tele* OR electr*) N2 (medicin* OR monitor* OR care* OR rehabilitation* OR

device*)) OR (tele* N2 health*) OR (electr*-health* NOT (electr*-health-record*)) OR mHealth OR m-Health* OR mobile-health* OR internet* OR website* OR digital* OR world-wide-web OR web-based OR webbased OR ((web OR mobile) N2 (application* OR app* OR device*)) OR ((mobile OR cell* OR smart*) N1 (phone*)) OR smartphone* OR virtual-realit* OR simulat* OR augment-realit* OR (electr* N2 system*) OR ((virtual*) N2 (care* OR communicat* OR technolog*)) OR tablet* OR (electr* N2 decision-aid*) OR smartwatch*)) **AND** (MH "Critical Care" OR MH "Intensive Care Units"+ OR MH "Critical Illness" OR TI (ICU OR GICU OR ((intensive OR critical*) N2 (care*)) OR critical*-ill*) OR AB (ICU OR GICU OR ((intensive OR critical*) N2 (care*)) OR critical*-ill*)) **AND** (MH "After Care" OR TI (aftercare* OR after-care* OR followup* OR follow-up OR ((post OR after) N2 (IC OR ICU OR intensive-care* OR discharge*)) OR postIC OR postICU OR postintensive* OR transition* OR ((ICU OR IC OR intensive-care* OR critical*-ill*) N2 (survivor* OR rehabilitat*)) OR mobili*) OR AB (aftercare* OR after-care* OR followup* OR follow-up OR ((post OR after) N2 (IC OR ICU OR intensive-care* OR discharge*)) OR postIC OR postICU OR postintensive* OR transition* OR ((ICU OR IC OR intensive-care* OR critical*-ill*) N2 (survivor* OR rehabilitat*)) OR mobili*))

Web of Science

TS=(((telehealth* OR tele-health* OR telemedicine* OR telemonitor* OR telecare* OR telerehabilitat* OR tele-rehabilitat* OR eHealth OR e-health* OR telerehabilitation* OR ((tele* OR electr*) NEAR/2 (medicin* OR monitor* OR care* OR rehabilitation* OR device*)) OR (tele* NEAR/2 health*) OR (electr*-health* NOT (electr*-health-record*)) OR mHealth OR m-Health* OR mobile-health* OR internet* OR website* OR digital* OR world-wide-web OR web-based OR webbased OR ((web OR mobile) NEAR/2 (application* OR app* OR device*)) OR ((mobile OR cell* OR smart*) NEAR/1 (phone*)) OR smartphone* OR virtual-realit* OR simulat* OR augment-realit* OR (electr* NEAR/2 system*) OR ((virtual*) NEAR/2 (care* OR communicat* OR technolog*)) OR tablet* OR (electr* NEAR/2 decision-aid*) OR smartwatch*)) **AND** ((ICU OR GICU OR ((intensive OR critical*) NEAR/2 (care*)) OR critical*-ill*)) **AND** ((aftercare* OR after-care* OR followup* OR follow-up OR ((post OR after) NEAR/2 (IC OR ICU OR intensive-care* OR discharge*)) OR postIC OR postICU OR postintensive* OR transition* OR ((ICU OR IC OR intensive-care* OR critical*-ill*) NEAR/2 (survivor* OR rehabilitat*)) OR mobili*))) *NOT (DT=(Meeting Abstract OR Meeting Summary) AND PY=1800-2019)*

Cochrane Central

((telehealth* OR tele NEXT health* OR telemedicine* OR telemonitor* OR telecare* OR telerehabilitat* OR tele NEXT rehabilitat* OR eHealth OR e NEXT health* OR telerehabilitation* OR ((tele* OR electr*) NEXT/3 (medicin* OR monitor* OR care* OR rehabilitation* OR device*)) OR (tele* NEXT/3 health*) OR (electr* NEXT health* NOT (electr* NEXT health NEXT record*)) OR mHealth OR m NEXT Health* OR mobile NEXT health* OR internet* OR website* OR digital* OR world NEXT wide NEXT web OR web NEXT based OR webbased OR ((web OR mobile) NEAR/3 (application* OR app* OR device*)) OR ((mobile OR cell* OR smart*) NEXT/1 (phone*)) OR smartphone* OR virtual NEXT realit* OR simulat* OR augment NEXT realit* OR (electr* NEAR/3 system*) OR ((virtual*) NEAR/3 (care* OR communicat* OR technolog*)) OR tablet* OR (electr* NEAR/3 decision NEXT aid*) OR smartwatch*):ab,ti,kw) **AND** ((ICU OR GICU OR ((intensive

OR critical*) NEAR/3 (care*)) OR critical* NEXT ill*):ab,ti,kw) **AND** ((aftercare* OR after NEXT care* OR followup* OR follow NEXT up OR ((post OR after) NEXT/3 (IC OR ICU OR intensive NEXT care* OR discharge*)) OR postIC OR postICU OR postintensive* OR transition* OR ((ICU OR IC OR intensive NEXT care* OR critical* NEXT ill*) NEAR/3 (survivor* OR rehabilitat*)) OR mobili*):ab,ti,kw)

Google Scholar

telehealth|telemedicine|telemonitoring|telecare|eHealth|e-health|telerehabilitation|mHealth|m-Health|'mobile|digital|web health|application|app|device' ICU|'intensive|critical care' aftercare|'after|post care|IC|ICU'|followup|'follow up'

Number of records

**Database searched**	**Platform**	**Years of coverage**	**Records**	**Records after duplicates removed**
Medline ALL	Ovid	1946 - Present	1219	1201
Embase	Embase.com	1971 - Present	2125	1318
Web of Science Core Collection*	Web of Knowledge	1975 - Present	1508	483
Cochrane Central Register of Controlled Trials**	Wiley	1992 - Present	346	118
CINAHL	EBSCO	1982 - Present	692	188
PsycINFO	Ovid	1806 - Present	63	5
Additional Search Engines: Google Scholar (200 top-ranked)			200	172
**Total**			**6153**	**3485**

*Science Citation Index Expanded (1975-present) ; Social Sciences Citation Index (1975-present) ; Arts & Humanities Citation Index (1975-present) ; Conference Proceedings Citation Index- Science (1990-present) ; Conference Proceedings Citation Index- Social Science & Humanities (1990-present) ; Emerging Sources Citation Index (2005-present)

** manually deleted abstracts from trial registries

Supplemental File S2 Description of the primary results

First author	Study groups (n)	Results
Capin[38]	Intervention: tele rehabilitation with videoconferencing (n=29)<br>Control: educational handout (n=15)	Primary outcome<br>- Adherence: consent 49/337 (14.5%), 44 were randomised; 27 of 29 participants (93%; 95% CI 77%–99%) receiving the intervention and attended ≥75% of sessions. Of the 29 participants receiving the intervention, 27 (93%; 95% CI 77%–99%) attended ≥75% of the sessions. Drop-out overall 4 (9.1%)<br>- Safety: No adverse events (AE) occurred during telerehabilitation sessions; 38% (11/29) of the intervention group compared with 60% (9/15) of the control group experienced a moderate or minor adverse event (p=.21)<br>- Fidelity: score of 99% with video recording of one treatment session for 23 participants<br>- Usability: 26 (93%) participants with median (IQR) score 72 [61, 75]<br>Secondary outcomes<br>- Both groups demonstrated improvement in exercise capacity, function and balance, balance confidence, breathlessness, depression, loneliness, self-efficacy, frailty, cognitive function but without statistically significant differences between groups
Cox[39]	Three arms:<br>1) Self-directed mobile app based mindfulness programme (n=31)<br>2) Therapist-led telephone based mindfulness programme (n=31)<br>3) Web-based education programme (n=18)	Primary outcome<br>- Feasibility: consent 74%, randomisation 91%, mobile app group 31 patients, 24 received intervention, 22 completed all sessions (92%), dropout overall 14 (17.5%)<br>- Acceptability: mean score 27.9 (SD 3·7); mobile mindfulness 27.6 (SD 3.8); telephone mindfulness 29.4 (SD 3.3) and education programme 25.7 (SD 3.2); p<.0001<br>- Mean usability score was 86.5 (SD 13.3) for the mobile mindfulness group (other groups not measured)<br>Secondary outcomes<br>- Clinically significant changes on anxiety, depression, physical symptoms. Post-traumatic stress, quality of life with the visual analogue scale, mindfulness skills and coping skills did not change
Cox[40]	Three arms:<br>1) Blueprint (BP) with therapist (n=16)<br>2) Blueprint without therapist (n=14)<br>3) Usual care (n=15)	Primary outcome<br>- Feasibility and adherence: eligible 210 patients, consent 63 (30.8%) patients; observed rates were similar to target feasibility benchmarks, including consented patients who were randomised (71.4%), adherence (97%). Dropout overall 11 (24.4%) and highest in the group without a therapist 6/14 (42.9%)<br>Secondary outcomes<br>- The intervention performed similarly on secondary outcomes whether a therapist was included or not in its effect on depression, anxiety, and post-traumatic stress symptoms as well as quality of life but improved for anxiety and PTSS compared to usual care.
Dong[42]	Intervention: cognitive rehabilitation training (n=68)	Primary outcome<br>- Cognitive function score intervention group was significantly higher than that in the control group (26.69 ± 2.49 vs 23.03 ± 3.79)<br>Secondary outcomes<br>- Quality of life improved in all areas in the intervention group

	Control: same except for cognitive rehabilitation training (n=68)	
Gerber[44]	Virtual Reality Cognitive Stimulation (n=57)	- Acceptance: usability mean 3.57 (range 0–4); immersion mean 2.70 (range 0–4); and satisfaction mean 3.13 (range 0–4); Lost to follow-up overall 7 (17.5%)<br>- Eye movements: number of gazed meaningful objects per minute was significantly lower during the ICU session than pre- and follow-up sessions, mean duration of fixation on meaningful moving objects did not differ between the sessions<br>- Discomfort during stimulation such as disorientation oculomotor problems and nausea were low<br>- Recollection of VR was high [28/33 patients (84.8%)], while recollection of ICU stay was low [10/33 patients (30.3%)]<br>- Quality of life: pre-ICU (mean 78, SD 13.27) and follow-up sessions (mean 85.24, SD 9·52) increased by 7.03 (95% CI 1.26–12.81, p<.019)<br>- Cognitive functions: pre-ICU (mean 27.52, SD 2.01) and follow-up sessions (mean 27.56, SD 2.02)<br>- Relaxing effect VR: no significant difference between the three sessions
Howroyd[45]	Homebased rehabilitation programme (n=38)	Primary outcome<br>- Utilisation of the virtual rehabilitation programme; including recruitment, retention, and adherence rates: thirty-eight of 76 eligible patients (50%) agreed to participate, of which 28 (74%) completed the rehabilitation programme<br>Secondary outcomes<br>- Exercise capacity perceived breathlessness, shoulder disability, anxiety, depression, psychological distress, quality of life all significantly improved pre- vs post rehabilitation following completion of the programme. No adverse events or injuries occurred
Hunter[46]	1) Smartwatches with multidisciplinary team (MDT) (n=14)<br>2) Smartwatches without MDT (n=21)	- Adherence, 35 (70%) used and uploaded data from their smartwatches. Complete data set at 1 year, 12 (24%)<br>- Daily step counts: increased from 4359 (SD 3488) in the first month following discharge to 7914 (SD 4146) steps per day at 1 year (P=.003). The MDT subgroup's mean step count increased more than the control group (176% increase vs 42% increase, respectively; +5474 steps vs +2181 steps, respectively; p=.04) over 1 year<br>- Daily resting heart rates decreased from 79 (SD 7) in the first month to 69 (SD 4) at 1 year following discharge (p<.001)
Petrinec[48]	Period 1) Usual care (n=25)<br>Period 2) Cognitive behavioural therapy by smartphone app (n=24)	- Feasibility and app use: consent 75%; completion rates study measures: mobile health group 79% vs control group 92%. Family members logged in to the app on average 18.58 times (range 2-89) during 81.29 minutes (range 4.93–426.63 minutes)<br>- Satisfaction: mobile health group mean rating 3.68 out of 5 (median 4)<br>- Mental health self-efficacy: increased in mobile app group, did not in control group<br>- Post-traumatic stress symptoms and health-related quality of life: no change in either group
Ramalingam[50]	Rehabilitation with video conferencing and apps (n=1)	- Only results at admission vs at discharge rehabilitation unit (the digital component of the intervention was applied at home, after discharge rehabilitation unit)

Reck[51]	Intervention: partner assisted cognitive-behavioural writing therapy; dyads (n=4), patients only (n=6), spouse only (n=2)<br>Control: waiting; dyads (n=5), patients only (n=6), spouse only (n=2)	- Post-traumatic stress symptoms: significant decrease before vs after writing therapy (33.2 (SD 13.12) vs 23.96 (SD 13.98); *d*=-2.16, 95% CI [-2.78 to 1.55]). No correlation time start writing therapy (immediately or delayed) and decrease PTSS<br>- Therapeutic alliance No correlation was found between treatment success and therapeutic alliance<br>- Exposure and cognitive restructuring: written words was 4143 (SD 2908), the more words written, the more reduction in PTSS (r=-0.41), 95% CI [0.06–0.76], p=.042
Rose[52]	5 patients using the pathway for 2 months (ongoing study).	- Acceptability and feasibility: 51 patients were eligible, 19 patients were enrolled (5 declined participation, 26 discharged before enrolment). The first five patients were described in this early-stage report<br>- Emotional recovery: All five patients identified anxiety, difficulty sleeping, fatigue and global weakness as barriers to recovery<br>- Health related quality of life: Short-term goals primarily focused on ability to walk independently with distance, time and amount of support personalised to the patient's ability. Medium term goals also focused on mobility with others related to activities of daily living such as preparing a meal and shopping. Educational resources were; returning to driving, coping with fatigue, nutrition at home, pain support, difficulty sleeping
Scruth[53]	4 families (patients and relatives)<br>Electronic ICU diary	- Feasibility and usability: The diary is useful for patients who cannot remember their ICU-stay, for communicating the patient's status to family members, to support family members in patient care conversations and giving family members a sense of control. Challenges were potential patient privacy violations and securing the tablet at the patient's bedside
Stromberg[54]	Intervention: Kundalina tele-yoga sessions in a group and an app with yoga instructions (n=5)<br>Control: active control (n=5)	Primary outcome<br>- Composite endpoint of physical function, health-related quality of life and symptoms of anxiety and depression: the intervention group showed some improvements in the composite-end point compared to the control group<br>Secondary outcomes<br>- Gait speed, sit-to-stand test health-related quality of life, sleep and cognitive function were described per patient<br>- Fidelity and adherence: Consent 10/25 (40%). All participants reached the goal of number of sessions to complete. One participant reached the goal of minutes used the app. Four participants in the control group followed their activity advice<br>- Exercise motivation: Two participants in the intervention group and one in the control group increased their total exercise motivation<br>- Acceptability: Intervention group, one experienced it as excellent, three as very good and one as good. Control group one found it very easy to use and four easy to use. No adverse events or injuries occurred
Taylor[55]	Intervention: additional sepsis transition and recovery (STAR) support (n = 349)	Primary outcome<br>- Composite outcome of mortality or hospital readmission at 30 days; 28.7% vs 33.3%; risk difference, 4.7%; odds ratio, 0.80; 95% CI, 0.58–1.11; adjusted odds ratio, 0.80; 95% CI, 0.64–0.98). There were 74 deaths (STAR: 33 [9.5%] vs UC: 41 [12%]) and 155 rehospitalisation's (STAR: 71 [20.3%] vs UC: 84 [24.6%])

	Control: usual care (UC) (n = 342)	Secondary outcomes<br>- All-cause mortality/hospital readmissions did not differ significantly between groups<br>- Process measures intervention implementation: nurse navigators contacted patients a median of 15 times for a total of 170 minutes: a care alignment tool was documented in the electronic health record for STAR 42% vs UC 23% (p<.01)<br>- Outpatient referrals: depression screening STAR 55% vs UC 10% (p<.01)<br>- Documented medication reconciliation: STAR group 76 % vs UC 50% (p<.01)
Tsavourelou[56]	451 patients were screened for participation in a homebased rehabilitation program	- Patient eligibility: 451 patients were screened for eligibility on mechanical ventilation > 48h; Mini Mental Examination Score >23/30; and a Rivermead Mobility Index >8/15: 55 patients were considered eligible<br>- A cost-benefit analysis showed that the initial investment leads to better financial output than re-hospitalization caused by incomplete rehabilitation
Vlake[57]	Intervention: ICU-VR (n=45)<br>Control: standard care (n=44)	Primary outcomes<br>- Prevalence and severity of psychological distress were limited throughout follow-up, no differences in psychological distress or quality of life were observed between the groups.<br>Secondary outcomes<br>- ICU-VR improved satisfaction with and rating of ICU aftercare and 81% of the patients thought VR improved the quality of aftercare
Wang[60]	Intervention: virtual reality-based intensive psychological intervention (VR-IPI) (n=56)<br>Control: Traditional psychological counselling (TPC) (n=50)	Primary outcomes<br>- Post-traumatic stress symptoms: baseline VR-IPI vs TPC (72 vs 70); 12 months (64 vs 18), p=.012<br>- Anxiety and depression: baseline VR-IPI vs TPC (7.65 vs 7.54); 12 months (2.87 vs 6), p=.032<br>- Negative Emotion: baseline VR-IPI vs TPC (7.9 vs 8); 12 months (2.3 vs 6.23), p=.043)<br>- Fear of ARDS recovery: baseline VR-IPI vs TPC (7.5 vs 7.4); 12 months (2.7 vs 6), p=.024<br>Secondary outcomes<br>- All the p-values for patient experience factors like feasibility, satisfaction, usability were statistically significant. VR-IPI is feasible and easier to use than TPC<br>- VR-IPI group exhibited significantly better scores for health, sleep quality, emotional stability, and physical activity than in TPC group
Wilson[62]	30 patients received computerized cognitive rehabilitation	- Measures of cognitive ability: improvement on some measures of cognitive ability (Digit Span, p=.06; Spatial Span, p=.05; Trail Making Test A, p=.05; Trail Making Test B, p=.05; Trails B-A [cost], p=.05)<br>- Improvement positively correlated with number of training hours, [F(1.22) = 3.0; *p*=.097]<br>- Eligible 113 patients, consent 30 (27%) patients; 24 patients completed the study
